# Prognostic implications of *PIK3CA* amplification in curatively resected liposarcoma

**DOI:** 10.18632/oncotarget.8240

**Published:** 2016-03-21

**Authors:** Joo Hoon Kim, Jae Seok Lee, Eo Jin Kim, Kyu Hyun Park, Ki Hyang Kim, Seong Yoon Yi, Han Seong Kim, Yong Jin Cho, Kyoo-Ho Shin, Joong Bae Ahn, Hyuk Hu, Kyung Sik Kim, Young Deuk Choi, Sunghoon Kim, Young Han Lee, Jin-Suck Suh, Sung Hoon Noh, Sun Young Rha, Hyo Song Kim

**Affiliations:** ^1^ Division of Medical Oncology, Department of Internal Medicine, Yonsei Cancer Center, Yonsei University College of Medicine, Seoul, Korea; ^2^ Department of Pathology, Dongguk University College of Medicine, Dongguk University Ilsan Hospital, Goyang, Korea; ^3^ Cancer Metastasis Research Center, Song Dang Institute for Cancer Research, Yonsei University College of Medicine, Seoul, Korea; ^4^ Department of Internal Medicine, Busan Paik Hospital, Inje University College of Medicine, Busan, Korea; ^5^ Department of Internal Medicine, Ilsan Paik Hospital, Inje University College of Medicine, Ilsan, Korea; ^6^ Department of Pathology, Ilsan Paik Hospital, Inje University College of Medicine, Ilsan, Korea; ^7^ Department of Orthopedic Surgery, Yonsei University College of Medicine, Seoul, Korea; ^8^ Department of Surgery, Yonsei University College of Medicine, Seoul, Korea; ^9^ Department of Urology, Yonsei University College of Medicine, Seoul, Korea; ^10^ Department of Obstetrics and Gynecology, Yonsei University College of Medicine, Seoul, Korea; ^11^ Department of Radiology, Yonsei University College of Medicine, Seoul, Korea

**Keywords:** liposarcoma, PIK3CA, amplification, mutation

## Abstract

**Background:**

We investigated the epidemiologic characteristics and prognostic significance of *PIK3CA* mutations/amplifications in curative resected liposarcoma.

**Patients and methods:**

A total of 125 liposarcoma tissue samples were collected over a 12-year period. *PIK3CA* mutations and gene copy number amplifications were analyzed by pyrosequencing and fluorescence in situ hybridization (FISH).

**Results:**

Nine of the 105 liposarcomas (8.6%) had activating *PIK3CA* mutation. *PIK3CA* mutations were more frequent in myxoid/round cell and pleomorphic tumors compared with well-differentiated/dedifferentiated tumors (13.3% vs. 2.2%, P=0.043). In FISH *PIK3CA* analysis, copy number gain was detected in 14 of the 101 tumors (13.9%): 11 (10.9%) tumors had increased gene copy number (polysomy) and 3 (3.0%) exhibited gene amplification. In survival analysis, patients with *PIK3CA* copy number gain had a worse prognosis compared to patients without *PIK3CA* amplification (median disease-free survival [DFS] 22.2 vs. 107.6 months p=0.005). By multivariate analysis, *PIK3CA* copy number gain was an independent prognostic factor for worse DFS (P=0.027; hazard ratio, 2.400; 95% confidence interval 1.105 to 5.213). *PIK3CA* mutation was not associated with DFS and overall survival.

**Conclusions:**

We demonstrated *PIK3CA* mutation and amplification in liposarcoma. *PIK3CA* copy number gain was an independent poor prognostic factor for DFS. Further studies are needed to evaluate the potential diagnostic and therapeutic role of *PIK3CA* mutations and amplifications in liposarcoma.

## INTRODUCTION

Soft tissue sarcoma (STS) is a heterogeneous disease with over 50 histologic subtypes, with various biological behaviors and genetic features [1]. In adults, liposarcoma is the most common type STS, representing 17–25% of the total cases [2]. Although surgical resection is the primary treatment for localized disease, many liposarcomas eventually progress to advanced disease that is either unresectable, metastatic, or both. For patients with those tumors, the mortality is high, and local and/or systemic tumor burden also results in significant morbidity [3].

For prognostic reasons, liposarcoma is subdivided into 5 subtypes by the World Health Organization classification: well-differentiated, myxoid/round cell, dedifferentiated, pleomorphic, and mixed-type liposarcoma [4]. Well-differentiated and myxoid liposarcoma are low-grade tumors, while dedifferentiated, round cell, and pleomorphic liposarcoma are high-grade tumors. Low-grade tumors have a low frequency of metastasis, and high-grade tumors often manifest with clinically aggressive behavior and distant metastasis. Besides the recognition of specific histologic subtypes, better understanding of distinct genetic and molecular aberrations is critical for therapeutic strategy.

The phosphatidylinositol-3-kinase (PI3K)-Akt-mTOR signaling pathway plays a central role in regulating tumor cell metabolism and survival [5]. Activation of the PI3K pathway occurs upon engagement with mutated or amplified phosphatidylinositol-4,5-bisphosphate 3-Kinase, catalytic subunit Alpha (*PIK3CA)* gene It increases p110α expression, PI3K activation, and phosphorylation of downstream signaling molecules. Akt-*PIK3CA* gene amplification was found in 10–30% of non-small cell lung cancer, breast cancer, and colon cancer [6–8]. Activating somatic mutations were also identified in various solid tumors [9, 10]. Recently, *PIK3CA* mutation was reported in 12% and 18% of myxoid/round-cell liposarcoma, and it was associated with Akt activation and poor clinical outcome [11, 12]. Despite accumulating evidence of biological role, there have been few studies reporting the frequency of *PIK3CA* aberration (including mutations and amplifications) for all liposarcoma subtypes.

In this study, we evaluated the frequency of *PIK3CA* amplification and mutation in surgically resected liposarcoma. Furthermore, we also determined the prognostic impact of *PIK3CA* genetic aberration for liposarcoma patients.

## RESULTS

### Clinical and pathological features

A total of Korean 125 patients with liposarcoma who underwent curative resection were analyzed. The clinical characteristics such as gender, age, primary tumor site, histology, and tumor size are presented in Table 1. The median age at diagnosis was 52 years (range: 18–84 years), and male was predominant (62.4%). The median tumor size was 14.1 cm, and one-third of all tumors were over 15 cm. Primary tumors mainly occurred in the extremities (n=61, 48.8%), with the other sites being the retroperitoneum/intra-abdominal region (n=35, 28.0%), inguinal area/genital organ (n=10, 8.0%), and other sites (n=19, 15.2%). The distribution of histologic subtypes were as follows: myxoid in 52 patients (41.6%), well-differentiated in 45 patients (36.0%), pleomorphic in 10 patients (8.0%), dedifferentiated in 9 patients (7.2%), and round cell in 9 patients (7.2%).

**Table 1:** Patient characteristics based on *PIK3CA* amplification

Characteristics	All patients		Copy number gain (Polysomy + Amplification)*		Normal copy number		*P*^†^
	No.	%	No.	%	No.	%	
**Number of patients**	125	100	14	13.9	87	86.1	
**Age, years** **Median (range)**	52 (18-84)		61 (37-84)		50 (20-83)		**0.023**
**Gender**							0.487
**Male**	78	62.4	8	57.1	58	66.7	
**Female**	47	37.6	6	42.9	29	33.3	
**Tumor size, cm** **Median (range)**	14.1 (1.0-37.0)		18.5 (5.5-37.0)		12.25 (1.0-31.0)		**0.040**
0-5 cm	19	15.2	0	0	16	18.4	
5-10 cm	26	20.8	3	21.4	18	20.7	
10-15 cm	26	20.8	1	7.1	20	30.0	
>15 cm	46	36.8	8	57.1	28	32.2	
unknown	8	6.4	2	14.3	5	5.7	
**Histologic classification**							0.063
**Well- and De-differentiated**	**54**	**43.2**	**9**	**64.3**	**33**	**37.9**	
Well differentiated	45	36.0	7	50.0	30	34.5	
Dedifferentiated	9	7.2	2	14.3	3	3.4	
**Mixoid/Round cell, Pleomorphic**	**71**	**58.8**	**5**	**35.7**	**54**	**62.1**	
Myxoid	52	41.6	2	14.3	44	50.6	
Round cell	9	7.2	1	7.1	5	5.7	
Pleomorphic	10	8.0	2	14.3	5	5.7	
**Primary tumor site**							0.051
**Extremity**	**61**	**48.8**	**4**	**28.6**	**46**	**52.9**	
Lower extremity	55	44.0	4	28.6	43	49.4	
Upper extremity	6	4.8	0	0	3	3.4	
**Retroperitoneum/intraabdomen**	**35**	**28.0**	**8**	**57.1**	**18**	**20.7**	
Retroperitoneum	27	21.6	5	35.7	15	17.2	
Intraabdomen	8	6.4	3	21.4	3	3.4	
**Inguinal area & genital organ**	**10**	**8.0**	**1**	**7.1**	**8**	**9.2**	
**Other area**	**19**	**15.2**	**1**	**7.1**	**15**	**17.2**	
**Time to recurrence, months** **Median (range)**	22.0 (3.7-240.9)		13.8 (6.2-95.2)		34.4 (3.7-240.9)		

*Abbreviations: PIK3CA*, phosphatidylinositol-4,5-bisphosphate 3-Kinase, Catalytic Subunit Alpha;CEN3, centromere3

* *PIK3CA* amplification was defined as if one of the following criteria is fulfilled: (1) *PIK3CA*/CEN3 ratio is ≥ 2.0, (2) average number of PIK3CA signal per nucleus > 4.0

* *PIK3CA* polysomy were defined as average number of PIK3CA signal per nucleus 4.0 ≥ to > 2.0.

† χ^2^ test, Fisher's exact test, *t*-test or Mann-Whitney U test.

### PIK3CA amplification

**Figure 1:**
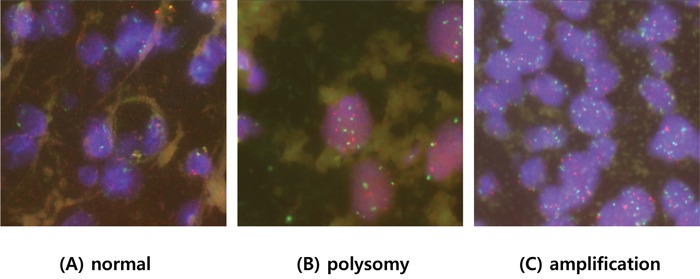
Representative fluorescent in situ hybridization of tumors with A. normal copy numbers, B. polysomy, C. and *PIK3CA* amplification

Of the 125 cases, 101 tumor samples were evaluable for *PIK3CA* FISH analysis. Based on *PIK3CA* gene expression values, tumors were categorized into three groups: (I) normal copy number, tumors with ≤2 copies per cell with a *PIK3CA*/CEN3 ratio < 2.0; (II) polysomy, tumors with an average *PIK3CA* signal per nucleus 4.0 ≥ to > 2.0; and (III) amplification, tumors with >4 copies per cell and/or a *PIK3CA*/CEN3 ratio ≥ 2.0. By these criteria, of the 101 cases, 14 tumors (13.9%) demonstrated copy number gain, 11 (10.9%) had polysomy, and 3 (3.0%) had gene amplification (Table 2, Figure 1). The median *PIK3CA* gene copy numbers were 2.3 (range, 2.1–2.6) and 6.5 (range, 5.0–10.3) in *PIK3CA* the polysomy and amplification groups, respectively. We also analyzed the association between *PIK3CA* amplification status and clinical parameters. *PIK3CA* copy number gain was significantly associated with older age and larger tumor size (Table 1, P=0.023 and P=0.040, respectively). There were no significant differences in *PIK3CA* copy number change with respect to sex, histology, and primary tumor site.

**Table 2:** *PIK3CA* amplification status according to liposarcoma histologic subtype

Histologic classification	Normal Copy Number	Copy Number Gain		
		Polysomy	Amplification	Total, n (%)
**Well- and De-differentiated**	**33**	**6**	**3**	**9 (21.4)**
Well- differentiated	30	6	3	
De-differentiated	3	0	0	
**Myxoid/Round cell, Pleomorphic**	**54**	**5**	**0**	**5 (8.5)**
Myxoid/ Round cell	49	3	0	
Pleomorphic	5	2	0	
**Total, n (%)**	**87 (86.1)**	**11 (10.9)**	**3 (3.0)**	
		**14 (13.9)**		

**Figure 2:**
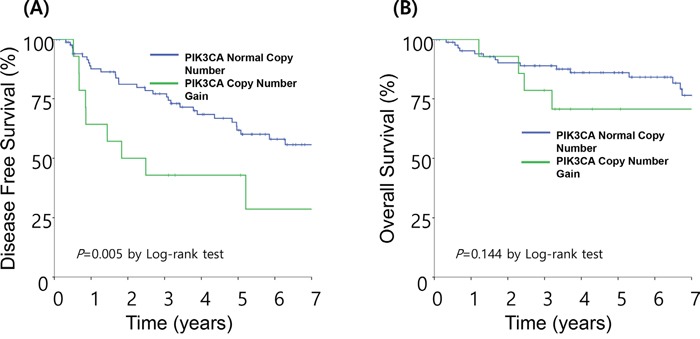
Survival analysis based on *PIK3CA* status . **A.** The median disease-free survival (DFS) was 22.0 months in patients with *PIK3CA* copy number gain and 107.6 months in the normal group. **B.** The median overall survival (OS) was not significantly different between the two groups.

With a median follow-up time of 50.6 months, the 5-year DFS and OS rates were 57.9% and 82.8%, respectively. The *PIK3CA* copy number gain group (polysomy and amplification) had a significantly shorter DFS compared with that of normal group (Figure 2A, median 22.0 vs. 107.6 months, P=0.005). Using the Cox proportional hazard model adjusted for gender, age, histology, and tumor location, *PIK3CA* copy number gain was an independent poor prognostic factor for DFS (Table 3, P=0.027; hazard ratio [HR], 2.400; 95% confidence interval [CI] 1.105 to 5.213). Older age (p=0.013; HR, 2.393; 95% CI 1.203 to 4.760) was also an independent poor prognostic factor for DFS. There was no significant difference in OS with respect to *PIK3CA* copy number gain (P=0.144, Figure 2B)

**Table 3:** Prognostic factors for disease-free survival and overall survival

Variable	Category	DFS			OS		
		HR	95% CI	*P*	HR	95% CI	*P*
***PIK3CA* copy number status**	Copy number gain *vs* Normal copy number (ref)	2.400	1.105-5.213	**0.027**	1.628	0.555-4.770	0.375
**Age**	Age ≥52 years *vs* Age < 52 years (ref)	2.393	1.203-4.760	**0.013**	3.875	1.351-11.118	**0.012**
**Gender**	Female *vs* Male (ref)	0.650	0.357-1.186	0.161	1.078	0.431-2.697	0.873
**Histology**	Mixoid/Round cell, Pleomorphic *vs* Well/De-diff (ref)	1.692	0.896 −3.196	0.105	1.336	0.538-3.316	0.533
**Primary tumor sites**	Extremity, Inguinal/genital, other area *vs* Retroperitoneum/Intraabdomen (ref)	0.880	0.439-1.762	0.718	1.335	0.420-4.242	0.624

*Abbreviations:* DFS, disease-free survival; OS, overall survival; HR, hazard ratio; CI, confidence interval; ref, reference; diff, differentiated

### PIK3CA mutation

**Figure 3:**
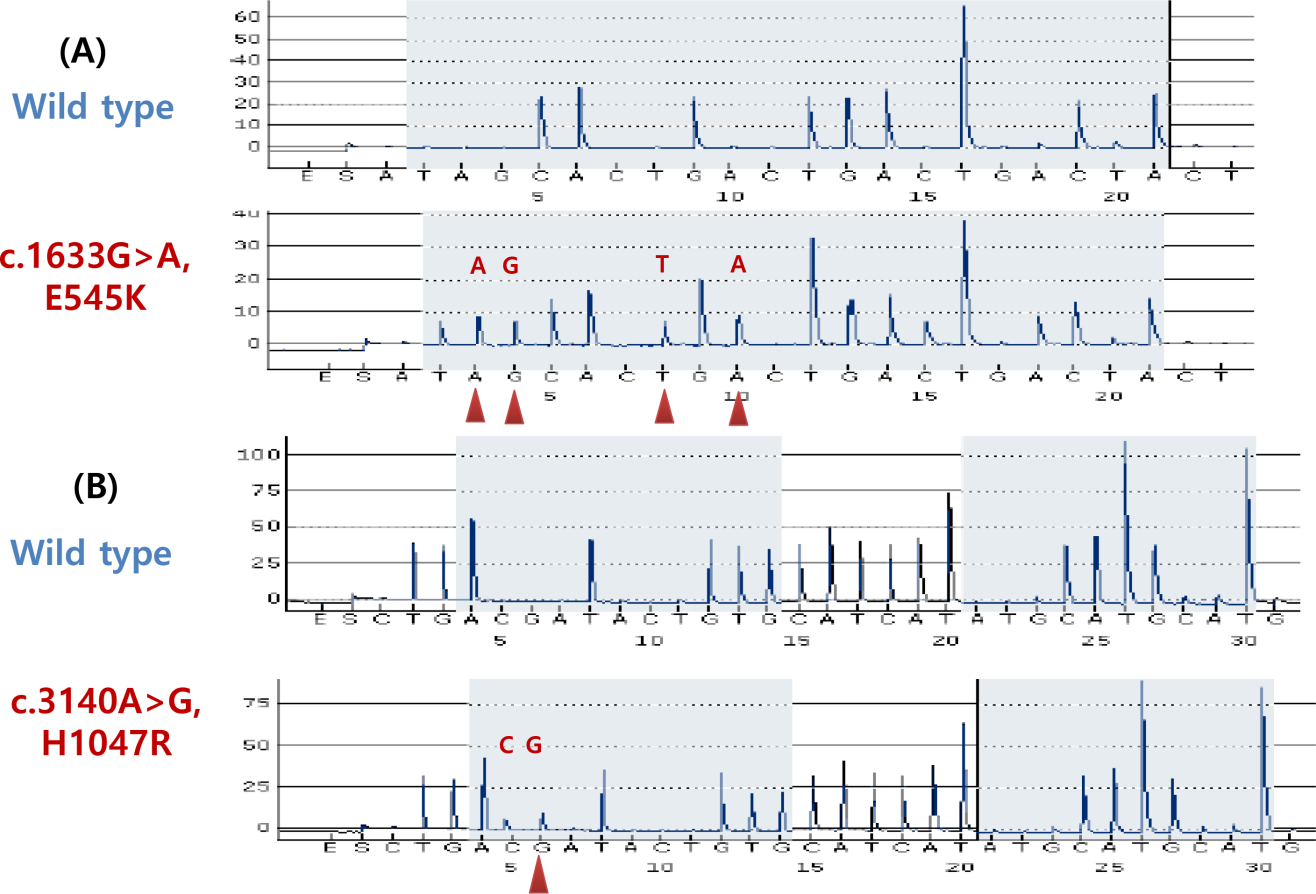
*PIK3CA* mutations in liposarcoma detected by pyrosequencing **A.** Wild-type and exon 9 E545K (G1633A) mutations. **B.** Wild-type and exon 20 H1047R (A3140G) mutations.

Among the 125 patients, 105 tumor samples were available for *PIK3CA* mutation analysis. Activating *PIK3CA* mutation was detected in 9 (8.6%) of 105 cases, with 5 cases of exon 9 mutation and 4 cases of exon 20 mutation (Table 4). For *PIK3CA* exon 9, E542K mutations were identified in 2 tumors, Q546K mutations were identified in 2 tumors, and an E545K mutation was identified in 1 tumor. For exon 20, H1047R mutations were identified in 3 tumors, and an H1047L was identified in 1 tumor (Figure 3). There was no co-occurrence of exon 9 and exon 20 mutations.

**Table 4:** *PIK3CA* mutational status according to liposarcoma histologic subtype

Histologic classification	Wild type	Mutation					
		Exon 9			Exon 20		Total, n (%)
		E542K	E545K	Q546K	H1047R	H1047L	
**Well- and De-differentiated**	44	0	0	0	1	0	**1 (2.2)**
Well- differentiated					1	0	1
De-differentiated					0	0	
**Myxoid and Round cell, Pleomorphic**	52	2	1	2	2	1	**8 (13.3)**
Myxoid and Round cell	43	2	0	1	2	1	6
Pleomorphic	9	0	1	1	0	0	2
**Total, n (%)**	96 (91.4)		5 (4.8)		4 (3.8)		*P*=0.043
				9 (8.6)			

With respect to histologic subtype, *PIK3CA* mutations were more frequent in well-differentiated/dedifferentiated tumors compared with myxoid/round cell/pleomorphic tumors (Table 4, 13.3% vs. 2.2%, P=0.043). Of all mutated tumors, 6 tumors (66.7%) were myxoid/round cell, 2 tumors (22.2%) were pleomorphic, and 1 tumor (11.1%) was well-differentiated (Table 4, Supplementary Table 1). There were no statistically significant differences in *PIK3CA* mutation based on sex, age, tumor size, and primary tumor sites (Supplementary Table 1). We also performed analyses to examine the association between *PIK3CA* mutations and survival outcome. *PIK3CA* mutation was not associated with a significant difference in DFS or OS (data not shown, p=0.739 and p=0.376, respectively)

### Correlation between PIK3CA mutation and copy number status

There were 82 cases with complete data on *PIK3CA* mutation and copy number status (Supplementary Table 2). Although not statistically significant, *PIK3CA* mutations were more frequent in the cases of copy number gain. Of the 7 tumors with *PIK3CA* mutation, 3 tumors (21.4%) exhibited copy number gain, whereas only 4 tumors (5.9%) had normal copy numbers.

## DISCUSSION

We conducted this study to examine the frequency and prognostic impact of *PIK3CA* mutations and amplifications in patients with curatively resected liposarcoma. To our knowledge, this is the first study evaluating the prognostic impact of *PIK3CA* polysomy and amplification in a large cohort of Asian liposarcoma patients. We demonstrated that *PIK3CA* aberration is a poor prognostic factor in liposarcoma.

Accumulating evidence has suggested different outcomes for the major histologic subtypes of liposarcoma. Therefore, histologic classification is known as a most strong prognostic factor for survival [13–16]. Among the 5 subtypes of liposarcoma, well-differentiated and myxoid liposarcoma had the most favorable prognoses, while dedifferentiated, round cell, and pleomorphic liposarcomas often manifest with clinically aggressive behavior and distant metastasis. Overall, with use of conventional doxorubicin-based chemotherapy, median survival remains less than 12 months for patients with metastatic/unresectable tumors [17]. The limited improvement after conventional therapy treatment prompts us to explore the molecular biology and identify prognostic and druggable biomarkers.

Despite known targeted therapeutic options in solid tumors (*KIT* or *PDGFRA* mutations in gastrointestinal stromal tumors), STS still lacks therapeutically relevant genetic alterations. Even knowledge of genetic alteration frequency is limited. A recent comprehensive genome study reported frequently mutated genes, including *TP53, NF1*, and *PIK3CA*, in STS [11]. Among them, *PIK3CA* mutation was observed in 18% of myxoid/round cell liposarcomas. Therefore, *PIK3CA* may be a putative driver gene in liposarcoma, with available therapeutic agents worthwhile.

Activation of the PI3K pathway, generally as result of *PIK3CA* amplification, has been demonstrated in 12% cases of lung cancer, 32% cases of head and neck cancer, and 24% cases of ovarian cancer [18–20]. Regarding the prognostic role of *PIK3CA* amplification, the results have been controversial. In head and neck squamous cell carcinoma, *PIK3CA* amplification led to earlier recurrence [18]. For nasopharyngeal carcinoma, amplification was associated with distant metastasis, advanced stage, and poor OS [21]. On the other hand, amplification played no significant role in squamous cell lung cancer cohort [8]. Clinicopathologic heterogeneity, including primary tumor site, pathologic stage, and adjuvant treatment, may contribute to these controversial results. However, because of its rarity of sarcoma, no exact frequency of *PIK3CA* amplification has been identified in sarcoma. Only The Cancer Genome Atlas (TCGA) data had reported 1% frequency of *PIK3CA* amplification and few comparable studies were reported with clinical cases. In our study, by carefully assessing a large cohort of liposarcoma cases, we were able to clarify the prognostic value of *PIK3CA* amplification in a homogenous patient population. *PIK3CA* amplification was significantly associated with poor DFS regardless of gender, age, histology, or tumor location, as an independent prognostic factor in curatively resected liposarcoma.

To evaluate *PIK3CA* copy number gain, we used FISH. Previous studies defined *PIK3CA/CEP3*≥2 [22–24] as *PIK3CA* amplification, and some studies used *PIK3CA*>4 [18, 25]. In our large cohort study, we used a combination of these criteria and demonstrated that amplification and polysomy have significant prognostic impacts on liposarcoma patients. FISH is an easy and useful method by which one can visualize individual cancer cells in routine clinical practice. Our criteria need further validation in future clinical trials with targeted agents.

Mutations in the *PIK3CA* gene are mostly located within hotspots in exons 9 and 20 and lead to PI3K pathway activation [26]. Regarding the prognostic significance of *PIK3CA* mutation, previous reports yielded controversial results [6, 26–29]. *PIK3CA* mutation has been reported 14–18.3% of myxoid/round-cell liposarcoma and associated with shorter DFS with mass spectrometry-based genotyping [11, 12]. Here, we report *PIK3CA* mutations in 8.6% of liposarcoma by employing a pyrosequencing approach and not associated with prognostic outcome. Although the majority of mutant tumors (66.6%) were of the myxoid/round-cell subtype, additional mutant cases were also detected in well-differentiated, dedifferentiated, and pleomorphic liposarcoma. The limited sensitivity of sequencing method may result in low frequency or clinical outcome and further studies are warranted.

Despite first-line standard treatment with doxorubicin, median survival remains dismal. gemcitabine and docetaxel are frequently used as second-line treatment for STS [30]. While the total response rate for all patients is 16%, only 2 out of 20 liposarcoma patients had stable disease at 6 months. Pazopanib was another potential breakthrough as a salvage treatment for STS [31]. However, pazopanib showed favorable efficacy in patients with leiomyosarcoma and synovial sarcoma, but not in adipocytic sarcoma [32]. Similar outcomes were observed in our Asian STS patients who received pazopanib treatment [33]. Therefore, exploration of therapeutically tractable target based on molecular biology is strongly warranted. Recently, local amplification of the 12q13-15 regions, which contain copies of *CDK4*, was reported in well-differentiated liposarcoma [34]. However, because that subtype is relatively indolent and easily curable with surgical resection, CDK4 inhibitors are not necessary for treatment of well differentiated liposarcoma [16]. In our study, we found frequent aberrations of the *PIK3CA* gene mainly in the aggressive subtypes (round cell and pleomorphic), and it is worth considering as a therapeutic target.

*PIK3CA* amplification and/or mutation are associated with increased activity of the PI3K effector pAkt, suggesting that amplified or mutated tumors may be sensitive to PI3K inhibitors [22, 35]. In lung cancer cell lines, 37% of squamous cell harbored *PIK3CA* amplification and they were sensitive to PI3K inhibitor GDC-0941 with less than 1μmol/L of IC50 [35]. In a preclinical platform from Cancer Cell Line Encyclopedia, *PIK3CA*-amplified tumors were sensitive to BYL719, a PI3K α-selective inhibitor [36]. Cell lines with *PIK3CA* amplification was positively associated with BYL719 sensitivity (P=0.0037) and tumor-bearing mice with *PIK3CA* amplification responded to BYL719, leading to a response rate of −18% (lung cancer) and −80% (gastric cancer). Despite preliminary data, recent phase I trials have explored the potential predictive role of the *PIK3CA* gene. In a phase I trial with GDC-0941, a heavily treated ovarian cancer patient with *PIK3CA* amplification experienced disease stabilization for 4 months with significant pharmacodynamic changes [37]. While previous trials with PI3K inhibitors have been conducted in non-selected patients, recent clinical trials with PI3K inhibitors are ongoing for those with *PIK3CA* gene alterations (ClinicalTrials.gov number NCT01928459 and NCT01608022). As shown in our study, liposarcoma may be a promising potential candidate for use of PI3K inhibitors. Further study with preclinical model is warranted to validate the therapeutic role in sarcoma.

Here, we report *PIK3CA* aberration as an independent poor prognostic factor for curatively resected liposarcoma. Our findings also indicate that *PIK3CA* inhibitor is a promising therapeutic target for liposarcoma.

## MATERIALS AND METHODS

### Patients and tumor tissues

This study was conducted in a cohort of patients with liposarcoma who underwent curative resection at Severance Hospital, Busan Paik Hospital, and Ilsan Paik Hospital. A total of 125 formalin-fixed, paraffin-embedded primary liposarcoma specimens were available for examination of *PIK3CA* aberrations. All diagnoses were reviewed by two experienced pathologists (LJS and HSK) in conjunction with immunohistochemical staining data. Only those cases of confirmed liposarcoma containing tissue adequate for analytic purposes were included. Liposarcoma was classified into 5 histologic subgroups based on Evans Classification [38]: well-differentiated, myxoid, dedifferentiated, round, and pleomorphic.

Patient information was collected by reviewing the medical records for evaluations of clinicopathologic characteristics and survival outcomes. Primary tumors were located in the extremity, retroperitoneum/intra-abdominal region, inguinal area/genital organs, and other.

### PIK3CA gene copy number analysis

To assess the presence of *PIK3CA* gene amplifications, fluorescent in situ hybridization (FISH) assays were performed using a *PIK3CA* probe that hybridizes to the band 3q26.32 with a Texas Red tag (red) and centromere3 (CEN3) with a FITC tag (green) (Abbott Molecular, Abbott Park, IL), according to routine methods. At least 60 nuclei were evaluated per sample. Twenty contiguous tumor cell nuclei were analyzed from three representative foci. *PIK3CA* amplification was defined based on previous studies [22, 23, 25, 39]. *PIK3CA* amplification was defined as if one of the following criteria is fulfilled: (1) *PIK3CA*/CEN3 ratio was ≥2.0 or (2) the average *PIK3CA* signal per nucleus >4.0. Polysomy was defined as an average *PIK3CA* signal per nucleus 4.0 ≥ to > 2.0.

### PIK3CA mutational analysis

Genomic DNA was extracted from 125 formalin-fixed paraffin-embedded (FFPE) tissue specimens using a QIAamp DNA FFPE Tissue Kit (Qiagen, Hilden, Germany), according to the manufacturer's instructions. We used a pyrosequencing assay covering the mutational hotspots of interest to sequence exons 9 and 20 of PIK3CA [40]. The exon 9 polymerase chain reaction (PCR) primers were PIK3CA 9-F, 5′-biotin-AACAGCTCAAAGCAATTTCTACACG-3′, and PIK3CA 9-R, 5′-ACCTGTGACTCCATAGAAAATCTTT-3′. The exon 20 PCR primers were PIK3CA 20-F, 5′-biotin-CAAGAGGCTTTGGAGTATTTCA-3′, and PIK3CA 20-R, 5′-CAATCCATTTTTGTTGTCCA-3′. Each PCR mix contained the forward and reverse primers (10 μM each), 12.5 mM dNTP mix, 3 mM MgCl_2_, 1× PCR buffer, 1 U of AmpliTaq Gold, and 100 ng of sample genomic DNA in a total volume of 25 μL. PCR conditions were as follows: initial denaturation at 95°C for 5 minutes; 50 cycles of 94°C for 30 seconds, 58°C for 30 seconds, and 72°C for 40 seconds; and final extension at 72°C for 10 minutes. The PCR products were electrophoresed in an agarose gel to confirm successful amplification. The PCR products were sequenced using the PyroMark Q24 system (QIAGEN, Germantown, MD, USA), according to the manufacturer's instructions. Sequencing analysis was performed using PyroMark Q24 software version 1.0.10 in the allele quantification analysis mode.

### Statistical methods

Associations between histologic features and clinical significance were examined using the χ^2^ test or Fisher's exact test where appropriate. Differences were statistically significant when the *P*-value was <0.05. Disease-free survival (DFS) was defined as the time from surgery to recurrence or last contact. Overall survival (OS) was defined as the time from surgery to death or last contact. Patients who were alive and had no recurrence at the last follow-up were censored. DFS and OS distributions were estimated using the Kaplan-Meier method. The log-rank test was used to determine survival differences between groups. Regression analyses of survival data, based on the Cox proportional hazards model, were conducted on DFS and OS. All data were analyzed using the Statistical Package for the Social Sciences Version 20.0 Software (SPSS Inc., Chicago, IL).

## SUPPLEMENTARY TABLES


