# Supplementary material for: Prognostic implications of PIK3CA amplification in curatively resected liposarcoma
Source: Oncotarget. 2016 Mar 21;7(17):24549–58. doi: 10.18632/oncotarget.8240 (PMC5029721; doi:10.18632/oncotarget.8240)
Supplement: Supplementary file 1 [file oncotarget-07-24549-s001.pdf]

SUPPLEMENTARY TABLES

Supplementary Table S1: Patient characteristics based on *PIK3CA* mutation

Characteristics	Mutation		No mutation		*P*
	No.	%	No.	%	
**Number of patients**	9	8.6	96	91.4	
**Age, years**<br>**Median (range)**	35 (31-66)		66 (18-84)		0.092
**Gender**					0.758
Male	6	66.7	59	61.5	
Female	3	33.3	37	38.5	
**Tumor size, cm**<br>**Median (range)**	6.0 (2.5-21.0)		14.0 (2.0-37.0)		0.227
**Histologic classification**					**0.043**
Well- and De-differentiated	**1**	**11.1**	**44**	**45.8**	
Well-differentiated	1	11.1			
De-differentiated	0	0			
Mixoid/Round cell, Pleomorphic	**8**	**88.9**	**52**	**54.2**	
Mixoid/Round cel	6	66.7			
Pleomorphic	2	22.2			
**Primary tumor site**					0.311
Extremity	4	44.4	41	42.7	
Retroperitoneum/intraabdomen	1	11.1	32	33.3	
Inguinal area & genital organ	1	11.1	8	8.3	
Other area	3	33.3	15	15.6	

Supplementary Table S2: Correlation of *PIK3CA* mutation with copy number status

	Copy number gain		Normal copy number		*P*
	**No.**	**%**	**No.**	**%**	
Mutation (n=7)	3	21.4	4	5.9	0.092
No Mutation (n=75)	11	78.6	64	94.1	
